# Advanced Polymeric Membranes as Biomaterials Based on Marine Sources Envisaging the Regeneration of Human Tissues

**DOI:** 10.3390/gels9030247

**Published:** 2023-03-20

**Authors:** Duarte Nuno Carvalho, Flávia C. M. Lobo, Luísa C. Rodrigues, Emanuel M. Fernandes, David S. Williams, Andrew Mearns-Spragg, Carmen G. Sotelo, Ricardo I. Perez-Martín, Rui L. Reis, Michael Gelinsky, Tiago H. Silva

**Affiliations:** 1 3B’s Research Group, I3B’s—Research Institute on Biomaterials, Biodegradables and Biomimetics of University of Minho, Headquarters of the European Institute of Excellence on Tissue Engineering and Regenerative Medicine, AvePark 4805-017, Barco, 4805-017 Guimarães, Portugal; 2 ICVS/3B’s—PT Government Associate Laboratory, 4710-057 Braga/Guimarães, Portugal; 3 Jellagen Limited, Unit G6, Capital Business Park, Parkway, St Mellons, Cardiff CF3 2PY, UK; 4 Group of Food Biochemistry, Instituto de Investigaciones Marinas (IIM-CSIC), C/ Eduardo Cabello 6, 36208 Vigo, Spain; 5 Centre for Translational Bone, Joint and Soft Tissue Research, Faculty of Medicine and University Hospital, Technische Universität Dresden, 01307 Dresden, Germany

**Keywords:** polymer–matrix composites (PMCs), thermal properties, mechanical testing, surface analysis

## Abstract

The self-repair capacity of human tissue is limited, motivating the arising of tissue engineering (TE) in building temporary scaffolds that envisage the regeneration of human tissues, including articular cartilage. However, despite the large number of preclinical data available, current therapies are not yet capable of fully restoring the entire healthy structure and function on this tissue when significantly damaged. For this reason, new biomaterial approaches are needed, and the present work proposes the development and characterization of innovative polymeric membranes formed by blending marine origin polymers, in a chemical free cross-linking approach, as biomaterials for tissue regeneration. The results confirmed the production of polyelectrolyte complexes molded as membranes, with structural stability resulting from natural intermolecular interactions between the marine biopolymers collagen, chitosan and fucoidan. Furthermore, the polymeric membranes presented adequate swelling ability without compromising cohesiveness (between 300 and 600%), appropriate surface properties, revealing mechanical properties similar to native articular cartilage. From the different formulations studied, the ones performing better were the ones produced with 3 % shark collagen, 3% chitosan and 10% fucoidan, as well as with 5% jellyfish collagen, 3% shark collagen, 3% chitosan and 10% fucoidan. Overall, the novel marine polymeric membranes demonstrated to have promising chemical, and physical properties for tissue engineering approaches, namely as thin biomaterial that can be applied over the damaged articular cartilage aiming its regeneration.

## 1. Introduction

Tissue engineering and regenerative medicine have made remarkable advancements in developing different temporary scaffolds capable of repairing or replacing damaged tissues resulting from trauma, degenerative pathologies or wear [1,2]. Many of these tissues, such as cartilage, have been considered particularly challenging to repair due to their lower regenerative capacity. In fact, being characterized by the absence of blood vessels and innervation, cartilage shows limitations in self-repair, especially in advanced clinical problems, e.g., tissue deterioration during ageing in the case of the hyaline cartilage tissue, or arthritis that encompasses a wide range of joint disorders, including both degenerative and inflammatory forms [3]. Cartilage tissue is composed essentially by a small percentage of chondrocyte cells, surrounded by a dense extracellular matrix (ECM) that enables diffusion of nutrients, gases and metabolism products, while preventing chondrocyte mobility during locomotion [4]. Typically, articular cartilage can be found covering some parts of bone surfaces to render low friction properties, reducing the wear and stress in the joint zones, thus facilitating the movements and absorbing/dissipating impacts resulting from mechanical shocks [5].

To overcome the regenerative process limitations, tissue engineering is exploring a range of methodologies and materials to manufacture biomaterials mimicking native ECM [6], namely porous scaffolds [7], hydrogels [8], cryogels [9], membranes [10] or 3D (bio)printed structures [11], among others. Those are selected according to their structural and biological properties and final approach. Ideally, these scaffolds should accomplish some basic principles for tissue engineering by: (i) showing specific biological properties such as nontoxicity, biocompatibility, low antigenicity and biodegradability, (ii) integrating well with the surrounding tissues; (iii) coping with the biomechanical stress, such as in locomotion; (iv) supporting the total integrity of cells and their viability that include proliferation and differentiation; and (v) promoting the natural ingrowth of the native tissue; among others [3,12]. In fact, biocompatibility is a critical concern and new scaffolds should cause only a negligible immune response and avoid severe inflammation, which is associated with a decreased healing rate and, in some cases, rejection by the human body [13].

Regarding scaffold manufacturing, membranes are a type of biomaterials, typically thin, that can be used to cover the surface of damaged tissues and organs. Currently, different types of methodologies are employed to manufacture this type of scaffolds, such as ionic or chemical gelation [14], solvent casting [15], electrospinning [16], decellularization of natural tissues [17] and by compaction of materials (e.g., ultracentrifugation) [18]. Furthermore, (semi)synthetic polymers and natural materials are suitable for the fabrication of membranes. In the last decade, marine-derived materials have gained considerable attention for biomedical application due to their similarities with many ECM components, low risks associated with zoonosis and overcoming social/religious-related constraints [12,19]. In this order, marine collagens have been considered great candidates for TERM strategies due to their biological properties such as high biocompatibility, low antigenicity, non-toxicity, safe biodegradability [20] and have the capacity to provide appropriate signals for cell adhesion, viability, proliferation and migration [20,21]. Additionally, chitosan has been used for tissue engineering in humans since it structurally shares a monomer with hyaluronic acid found in ECM, especially in cartilage tissues [22]. Being mainly obtained from chitin present in crustacean shells (i.e., shrimps and crabs) and squid pens, this polymer contains remarkable properties such as biodegradability, biocompatibility, low toxicity, anti-inflammatory and antibacterial, making it a potential candidate for cartilage tissue repair [23,24]. In the same way, fucoidan is commonly considered to resemble sulfated glycosaminoglycan (such as chondroitin sulfates) found in ECM [25]. Recent studies have demonstrated diverse biological properties for this sulfated polysaccharide commonly extracted from brown seaweeds, such as anticoagulant, anti-thrombotic, antiangiogenic, anti-inflammatory, antiviral and antioxidant, among others [26,27].

In an era where the exploitation of resources and the environmental impact of actions are under close scrutiny towards sustainability, the establishment of eco-friendly processes should be a goal on any technological development, including the production of scaffolds for tissue engineering. From one side, strategies of biomass valorization under the circular economy concept, i.e., sustainable exploitation of “blue resources” and the management of industrial by-products, should be addressed [10,28]. Likewise, the environmental costs of the proposed scaffolding process should be explored, by evaluating the waste generated during this process and accounting reagents, solvents and other reagents used. For this understanding, green metrics have been developed, such as the Environmental factor (*E*-factor) [29,30], which is a measure of the efficiency of a chemical reaction or process (including natural and synthetic materials) that takes into account the amount of waste generated in relation to the amount of product produced [31].

The present study aims to demonstrate the feasibility of manufacturing biomaterial-membranes formed by marine collagen (from jellyfish and shark skin), chitosan (from squid pens) and fucoidan (from brown algae) using natural ionic cross-linking. For this purpose, membranes were developed using different formulations comprising a combination of polymer ratios, and further characterized regarding their chemical and morphological features, surface properties, mechanical behavior and polymer distribution. Furthermore, this innovative approach was designed envisioning its future application in tissue engineering, including to cover the damaged cartilage surface, promoting its regeneration.

## 2. Results and Discussion

### 2.1. Green Metrics on Scaffolding Membrane Process

Currently, humanity has a growing concern regarding the sustainability of products and processes in diverse areas (for instance, pharmacological products or biomaterial processing in tissue engineering), equally for the use in the short or long term. In this way, it is necessary to apply green metrics such as the Environmental factor (*E*-factor) to evaluate how green the scaffolding process herein proposed is, considering the resource efficient extraction and the waste generated during the process (e.g., reagents and solvents). These metrics give the developers essential information and predictions about the materials’ impact when discarded [32,33]. Theoretically, when a higher *E*-factor is obtained, more waste is generated, and consequently the product has an undesirable impact on the environment, as so, preferably the *E*-factor needs to be closer to zero [29]. The *E*-factor was calculated to evaluate if the newly developed membranes could be considered safe for the environment. According to Sheldon equation [30], all the results obtained are very close to zero, between 0.11 to 0.17, which confirms that our biomaterials and the process adopted to their development can be considered sustainable for the environment due to their low impact, highlighting in addition that these biomaterials were produced with biodegradable components. Likewise, this scaffolding process explores a green methodology by using natural ionic cross-linking between marine polymers, i.e., electrostatic interactions that occur between the positively charged groups found in both collagens and chitosan polymeric chains (charged amines), and the negatively charged groups of the fucoidan chains (sulfates). In this order, it avoids the use of any chemical cross-linking agents, which may have a negative impact on the environment and, in some cases, be associated with some cytotoxicity effect to the cells [34].

According to this methodology, the aspects that can be considered less green for the environment are the use of acidic and basic solutions. They comprise hydrochloric acid (HCl) and sodium hydroxide (NaOH) at low concentrations during the polymers’ extractions and the ammonium acetate buffer (NH_4_OAc/AcOH) to dissolve both collagens and the chitosan during the biomaterial processing. Furthermore, E-factor studies can also be particularly helpful in determining whether it will be feasible to scale up a manufacturing process, including the polymeric extraction process. A manufacturing process efficiency may be affected when it is scaled up, which could result in more waste being produced and energy being used. Researchers can predict potential problems that might emerge during scale-up and fix them before they become a problem by conducting E-factor studies at a lesser scale. This can lessen the environmental impact and improve the commercial viability of tissue engineering materials by ensuring that the production process stays sustainable and effective as it is ramped up.

### 2.2. Determination of Glycosylation by Glycoprotein/Carbohydrate Estimation in Collagen Samples

Collagen is a natural protein that can be abundantly found in both invertebrate and vertebrate animals, which is essentially formed by polypeptide chains combined to form a right-handed triple helical coil, typically represented by Gly-x-y, where Gly is the amino acid glycine, and x and y are associated to other amino acids, with higher prevalence to proline (Pro) and hydroxyproline (Hyp) [12]. During the biosynthesis of these proteins, collagen acquires a specific number of co-translational or post-translational modifications, which are essential for their functional integrity. Those include the hydroxylation of Pro and Lys (lysine) residues, glycosylation of specific hydroxylysine (Hyl) residues that is indispensable for fibril formation mediated by β(1-O)galactosyl- and α(1-2)glucosyltransferase enzymes, and the cross-linking formation by covalent intra- and intermolecular bonds [35]. Furthermore, these modifications significantly increase the thermal stability of collagen, which is a crucial key to support the body temperature [36] and thus impact the performance of collagen-based biomaterials. To understand structural differences between the collagen samples (type II jCOL and type I sCOL), the glycosylation quantification was estimated using a glycoprotein carbohydrate estimation kit. The glycosylation process relies on chemical reaction in which a carbohydrate or ‘glycan’, i.e., a glycosyl donor, is attached to a hydroxyl or other functional group, and its quantification is based on the kit ability to estimate the presence of this type of bonds [37]. Taking this into account, the values estimated for jCOL are between 2.93 and 3.05%, while for sCOL they are between 6.34 and 9.04%. Furthermore, Thierry Hennet [38] explains that glycosylation varies according to the type of collagen due to the presence of the amino acid hydroxylysine on each molecular composition, being already reported that the collagen type IV is more extensively glycosylated when compared with collagen type II and type I. To summarize, our results suggest that the sCOL sample can be considered more structurally and thermally stable than the jCOL. These differences can provide a significant influence on the structural stability of the developed biomaterial systems.

### 2.3. ^1^H Nuclear Magnetic Resonance (^1^H-NMR) Analysis

**Figure 1:**
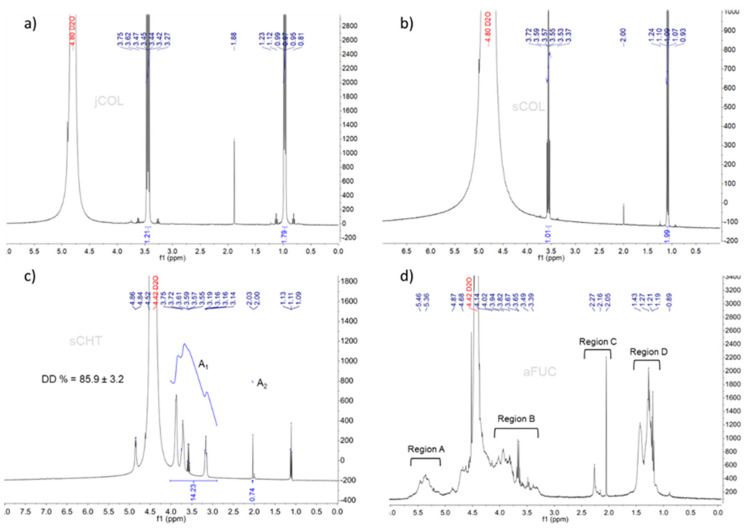
^1^H Nuclear magnetic resonance (^1^H-NMR) spectra of each marine polymer: (**a**) Collagen from jellyfish (jCOL); (**b**) Collagen from shark (sCOL); (**c**) Chitosan from squid pens (sCHT) and the respective degree of deacetylation (DD) and (**d**) Fucoidan from brown algae (aFUC).

^1^H-NMR spectral analysis is a remarkable technique that uses information regarding the hydrogen atom position in molecules to infer about their structure [39]. The ^1^H-NMR spectra of collagen from jellyfish (jCOL) and shark (sCOL) are both shown in Figure 1a,b, respectively. The results demonstrate a very intense band in both spectra at ~4.8 ppm, which indicates the presence of water molecules in the collagen samples. This water can be called ‘hydration’ water [40]. In optimum collagen preparation for this analysis, the absorbed water interacts with the collagen surface, helping to stabilize the collagen helix structure [41]. Moreover, singlet peaks can also be found at 1.12 ppm, 3.27 ppm and 3.62 ppm for jCOL and at 1.24 ppm, 3.37 ppm and 3.72 ppm for sCOL that indicates the unfolding amide proton, and α-carbon protons, while the chemical shifts founded at 1.23 ppm for jCOL and 1.24 for sCOL indicates the acid reacted of the proline (amino acid present in collagen repeat model). These spectra coincided with the ^1^H-NMR spectra of marine collagen analyzed by Krishnamoorthi et al. [39] and by Angilè et al. [42].

Additionally, the degree of deacetylation (DD) of the chitosan sample could be calculated by ^1^H-NMR spectroscopy using the integration peaks of the non-anomeric and anomeric protons resonance signals, shown in Figure 1c, corresponding to the ratio of deacetylated D-glucosamine units is respect to the total number of monomers [23]. The integral value of the broad A_1_ zone (protons in positions C2–C6 on the sugar ring) was 14.23, while for A_2_ zone (the three N-acetyl protons of GlcNAc) the integral value was 0.74, resulting in a DD value of 85.9% ± 3.2, which stands in line with the DD values presented in the literature for most of the reported chitosan samples [43,44,45]. This allowed to validate the innovative methodology proposed by the authors in order to obtain chitosan with medium to high DD using a low number of steps when compared with traditional production methods [46]. According to the literature, there is a direct correlation of the DD value with the chitosan physicochemical properties, such as solubility, degradation rate and hydrophilicity character, and therefore an expected increase in their DD values [47,48].

The ^1^H-NMR of fucoidan (sulfated polysaccharide) is very complex, as demonstrated in Figure 1d. In fact, the most common structure of fucoidan is composed by alternating linked (1-3) or (1-4)-α-L-fucopyranose (α-L-Fucp) and β-D-galactopyranose (β-D-Galp), which can present variations at C2 and C4 position, the structure being partially acetylated or sulfated [49]. Regarding this, it is possible to divide the achieved fucoidan spectra into four principal regions (A-D), disregarding the biggest peak at 4.4 ppm that is typical of the presence of residual water. The first broad signal observed at 5 to 5.7 ppm, named as region A, is associated to α-anomeric protons, being related with the presence of α-3 linked and α-3, 4 linked L-fucopyranose residues [50]. The second broad signal at 3.4 to 4.0 ppm (Region B) is attributed to the presence of ring protons of the galactose residues. However, if we admit the signal extension between the region C (2.0 to 2.3 ppm) and region B, this indicates the presence of CH_3_ protons of O-acetyl groups [51]. The last broad region signal (region D), appears at 1 to 1.5 ppm and represents the C6 methyl protons of L-fucopyranose (fucose residues) that confirms the presence of sulfated polysaccharide structure [52]. This result is in-line with the findings earlier reported for fucoidan structures from different seaweeds [53,54,55].

### 2.4. Chemical Characterization of Polymeric Membranes

**Figure 2:**
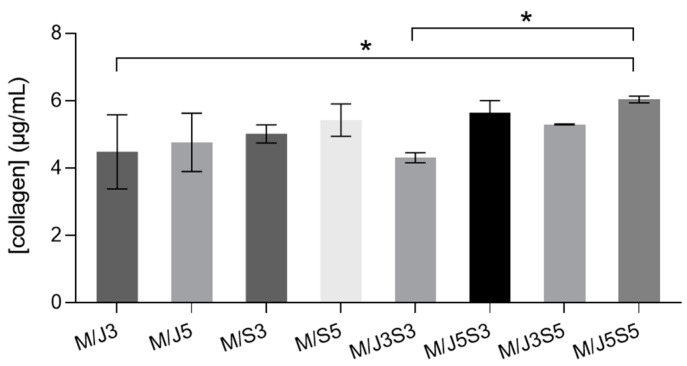
Quantification of total collagen concentration on developed membranes (M/J3 to M/J5S5). Data bars are mean ± standard error (n = 3). Comparative statistical analysis was performed, where systems M/J3, MJ3S3 and M//J5S5 include statistical significance of *p* < 0.05 (*), and ns (no significant) for the rest of systems.

The amounts of collagen present in collagen extracts produced from jellyfish and shark skin and in each developed membrane were quantified by enzymatic fluorimetric method. This simple and highly sensitive methodology consists of using enzymatic digestion of collagens into peptides, where the N-terminal glycine from the peptides would further react with the dye reagent to form a fluorescent complex. The fluorescence intensity of this product is directly proportional to collagen concentration in the sample. The total collagen concentration present on the marine membranes is shown in Figure 2.

Initially, comparing both collagen samples (jCOL and sCOL) individually, it is noticed that the concentration of collagen is significantly different between them, i.e., the sCOL contain almost 40% more collagen than jCOL. This discrepancy can be associated with the type of collagen, source, their amino acids’ sequential composition and extraction methods, among others [56], or even due to the sCOL demonstrating an easier ability to dissolve in the solvent. Furthermore, since the formulations (M/J3 to M/J5S5) were achieved after polymeric reticulation, this quantification was also assessed to observe the reticulation’s effect on the collagen’s ability to react within methods using enzymes. Thus, it was possible to verify the probability of having some collagen losses during the membranes’ processing. In this order, the total collagen content present on each biomaterial-membrane shows that the small concentration variations observed are mostly associated with the formulation since some samples contain more collagen per mL than others. As expected, the achieved data through this method are correlated with the prepared compositions (Table 2), being the formulations with higher initial amounts of collagen the ones with higher values of collagens. To demonstrate this fact, as an example, the formulation M/J5S5 presents the higher collagen concentration values, which is in accordance with the amounts of both collagen types used for the formulation preparation. Nevertheless, the values obtained for the developed biomaterials can be influenced by some minor errors as the collagens are combined with chitosan and fucoidan polymers, which impeded the process of dissolving the samples. Overall, the data obtained are in accordance with the % *w*/*w* of total polymer mass in the biomaterial-membranes extrapolated.

**Figure 3:**
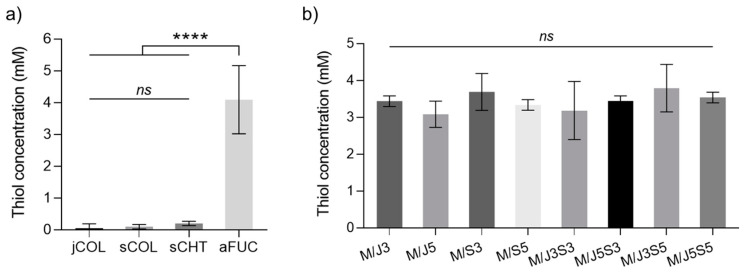
Determination of thiol (-SH) contents (**a**) determination on marine polymers (jCOL, sCOL, sCHT and aFUC), and (**b**) determination on the developed biomaterial-membranes (M/J3 to M/J5S5). Data bars are mean ± standard error (n = 3). The membrane samples do not show statistical differences represented by ns (no significance) except those represented with the symbol **** (*p* < 0.0001).

Ellman’s assay is a non-destructive method, which contains the Ellman’s reagent (5-5′-dithio-bis(2-nitrobenzoic acid)), also called DNTB, that is a chemical product used to form free sulfhydryl groups (free thiol groups) in solution-measuring methodology [57]. In general, DNTB solution reacts with free sulfhydryl molecules, producing a mixed disulfide and 2-nitro-5-thiobenzoic acid (TNB) product. Likewise, this method has the advantage that it can also be used to quantify these free groups in single polymers as well as in materials after processing, as the produced membranes [58]. The thiol groups were quantified in each marine polymeric solution, and of the developed membranes (data are shown in Figure 3).

The quantification of the thiol groups present in each developed membrane can be helpful, as it allows to estimate the real amount of fucoidan in these biomaterials and, consequently, assess the efficiency of the membrane scaffolding process. As expected, the availability of free sulfhydryl groups in marine biopolymer samples was observed, and its presence in the fucoidan sample was confirmed, while the rest of marine samples only demonstrated some insignificant residues. The value achieved for the fucoidan membrane will be further used as a control, as the same initial concentration was used to prepare the biomaterial-membranes. Furthermore, according to the present results, no significant differences were observed between samples and the control (fucoidan sample), proving that the proposed methodology is a sustainable approach for developing membranes based on marine sources without having significant losses of the fucoidan polymer during the processing. The minor variations observed between the membrane samples could be related to (i) the mass percentage (% *w*/*w*, see Table 2) of polymer in the biomaterial-membrane; and (ii) the reduced number of sulfate groups that remain available after polymerization, since they are involved in the intermolecular interaction established into the blended network.

**Figure 4:**
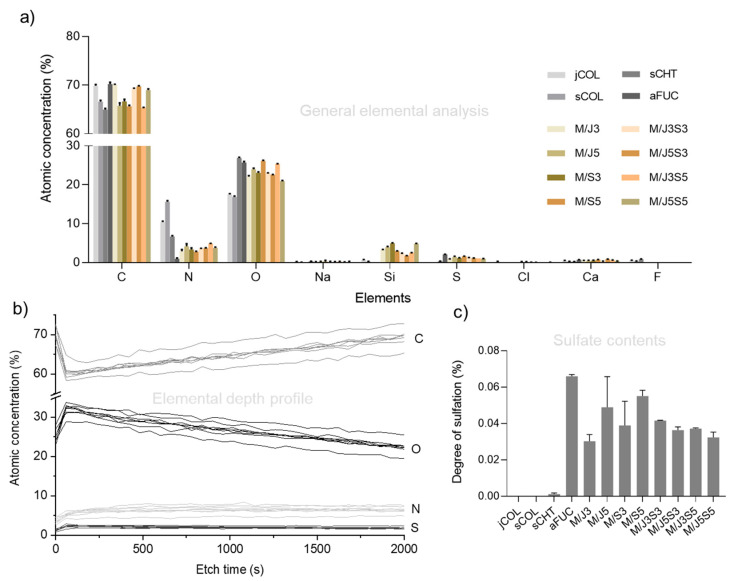
XPS analysis on the marine biopolymers and the developed membranes. (**a**) Atomic concentration (%) of main elements at the surface of the material; (**b**) atomic concentration (%) of main elements from in-depth profile assay as a function of etch time; and (**c**) determination of the sulfate contents (%) in all samples.

To further address the chemical composition of the produced polymeric membranes, X-ray photoelectron spectroscopy (XPS) was performed in different locations of the surface of each sample (considering an analysis depth of approximately 5 to 10 nm) and in-depth profile mode (Etch depth), with the purpose of investigating the spatial distribution of main chemical elements, particularly in a comparative way. All XPS data are summarized in Figure 4 and Table 1.

The data of elemental atomic concentration shown in Figure 4a were used to investigate the polymeric composition of each membrane surface. The evaluation of different locations allowed to ensure the homogeneity of the produced membranes surfaces. Some polymers contain specific elements that enable identification and quantification, as is the case of fucoidan, the only marine biopolymer used in this study containing sulfur (from sulfated groups) [55,59]. However, this strategy cannot be applied to distinguish biopolymers that contains similar elements in their composition, such as collagen and chitosan, the compositions of which are rich in carbon, oxygen and nitrogen [60,61]. The determination of sulfur/carbon (S/C), sulfur/nitrogen (S/N) and nitrogen/carbon (N/C) ratios, shown in Table 1, allowed to establish a more precise method to access the relative composition of the developed membranes. According to the data, the elemental composition of the membranes surface is very similar between the different compositions. All formulations containing fucoidan had lower sulfur/carbon ratio values in comparison with sulfur/nitrogen values, as expected from the used formulations (with C being a much more abundant element than N). Furthermore, only minor variations between the locations of each sample were appreciated, as expressed by the reduced standard deviations (SD) achieved. This can be considered a good indicator of the efficiency of the production method, rendering uniform biomaterials. In fact, the small detected variations between the samples can be directly related to the different initial polymer concentration used for biomaterial preparation (Table 2). Theoretically, a good polymer distribution plays a significant role in establishing analogous inter- and intra-chain bonds between the polymers, which directly influence the biomaterial microenvironment. Being also capable of increasing the structural stability in long-term, influence on polymer degradation likewise can act on cellular performance, such as on adhesion, distribution and proliferation of cells [12,62].

A supplementary XPS analysis was performed on each developed membrane to evaluate an in-depth profile (Etch depth) of the distribution of selected elements, with the results depicted in Figure 4b. It is possible to observe that the atomic concentration of carbon decreased after the first two etching cycles, while consequently an opposite profile was observed for the other elements, namely sulfur, oxygen and nitrogen. This occurrence may be due to the orientation of the functional groups, wherein the hydrophilic network functional groups orient towards the interior of the material. With the following etching cycles, a relative elemental composition similar to that detected at the surface was achieved. The atomic concentration of sulfur present in samples containing fucoidan was also used to assess the sulfate contents, shown in Figure 4c. In these results, statistical differences between the samples were not observed, which is in accordance with the results obtained in the thiol group’s quantification assay.

### 2.5. Physical Characterization of Polymeric Membranes

**Figure 5:**
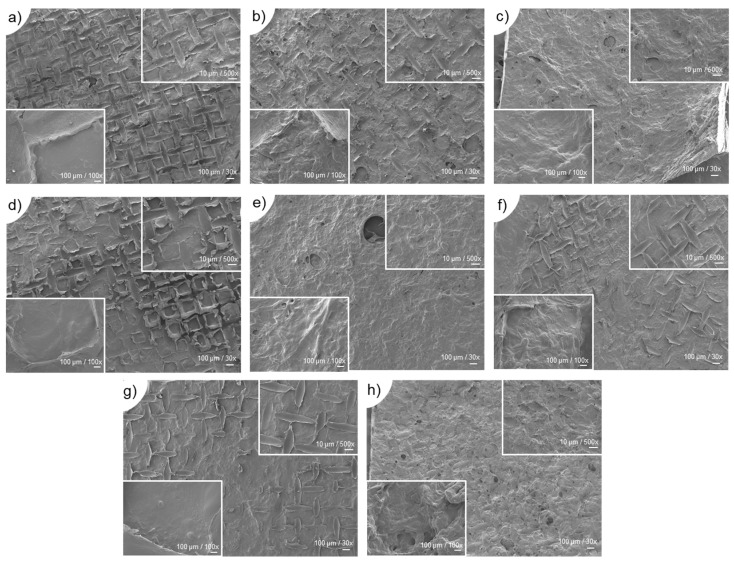
Scanning electron microscopy (SEM) images of the surface of all developed membranes (M/J3 to M/J5S5) according to the established formulations, (**a**–**h**), respectively. All samples were imaged at the magnifications of 30×/100 µm (**main**), 100×/100 µm (**bottom left**) and 500×/10 µm (**top right**).

Figure 5 illustrates the scanning electron microscopy (SEM) analysis of the surface morphology of the developed membranes.

According to the surface morphological characteristics exhibited by the developed membranes, no macro or micropores could be observed, but some roughness was present, together with “stretch marks” induced by the use of the nylon mesh on the disk molds to prepare the membranes. During the molding process, the polymeric blend suffered some pressure while the use of filter paper absorbed the excess of solvent, resulting in apparently compact structures. Nowadays, the scarcity of porosity in these type of structures is accepted by clinicians, considering such structures helpful to avoid excessive moisture in the lesion site and subsequent loss of the membrane physical properties in long-term, which determines the degradation time of the biomaterial and its effectiveness for tissue repair [63].

**Figure 6:**
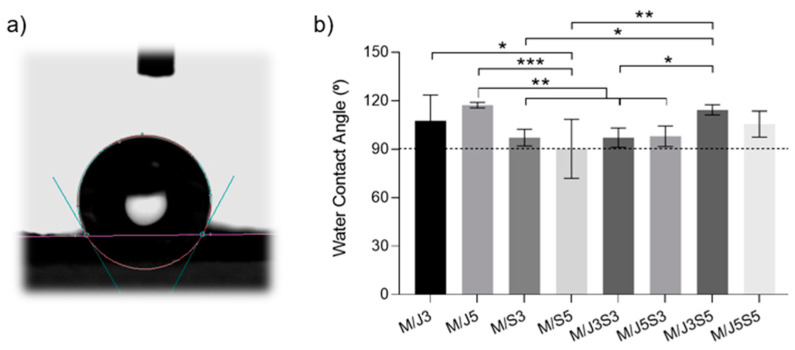
Water contact angle (WCA) measurements for the study of surface hydrophilicity on the polymeric membranes: (**a**) representative image of a water droplet deposited on the membrane surface, and (**b**) statistical analysis of the mean water contact angle obtained for the different membranes. Results are exhibited as mean ± standard error of three independent experiments. Statistical analysis of multiple comparison test (*p* < 0.05) was performed, showing a significance of * (*p* < 0.05), ** (*p* < 0.01), and *** (*p* < 0.001).

Water contact angle measurements were performed to test the surface wettability of the developed membranes [64]. The obtained data are shown in Figure 6.

In general, the contact angle property is determined by the attractive force of the droplet molecules on the surface (adsorption force) and the attractive force between the droplet molecules (cohesion). Therefore, when cohesion is more dominant than adhesion, the droplets will not easily wet the surface, being classified as hydrophobic surfaces [65]. In this order, when the contact angle is higher than 90°, the surface comprises hydrophobic properties, while when the angle is lower than 90°, the surface of the materials has hydrophilic properties. According to the results expressed in Figure 6b, all membranes exhibited water contact angle degrees higher than 90°, which indicates a hydrophobic nature. Higher values were registered for samples M/J5 (117.2° ± 1.6), and M/J3S5 (114.3° ± 3.1) though without significant statistical differences between samples. This surface hydrophobic behavior could impact the time required for biomaterial swelling. However, since the membranes were produced using hydrophilic polymers, their core might be able to preserve the material’s internal moisture for a longer time. This property would be helpful when envisaging application for cartilage repair since this tissue requires a constant high water content to maintain low friction during human body locomotion [66].

**Figure 7:**
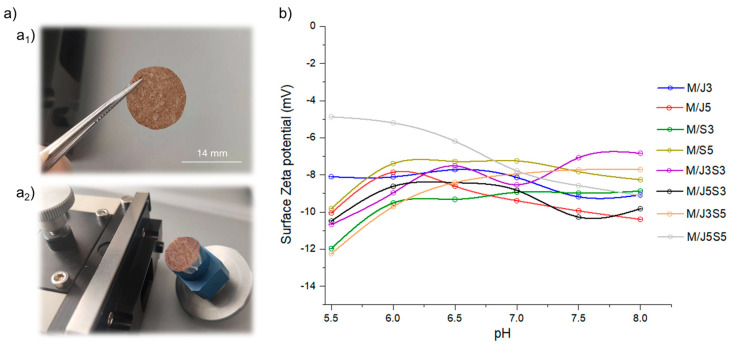
Surface Zeta (ζ) potential measurements on the membranes M/J3 to M/J5S5: (**a**) digital image of one representative membrane sample (M/J3), being glued to one surface of the surpass container, where (**a_1_**) illustrates the situation before the measurement and (**a_2_**) after used; and (**b**) titration curves of surface zeta potential within the pH range of 5.5 to 8.

The water contact angle is a surface property related with the surface energy of the material, also dependent on the surface charge, which can be assessed by the determination of the ζ-potential (also known as electrokinetic potential). This surface property can be obtained using the Helmholtz-Smoluchowski equation, where the streaming potential is generated by particles in circulation (electroosmotic flow) due to a differential pressure that can be measured using a voltmeter equipped on SurPASS electrokinetic analyzer. The data of ζ-potential obtained within the pH range of 5.5 to 8 are demonstrated in Figure 7.

All the analyzed membranes revealed a negative zeta potential, with the obtained absolute value not being significantly high, in coherence with the water contact angle obtained (in the hydrophobic region). Fucoidan is a negatively charged polymer, and it is the one that, in general, is in higher concentration in the membranes, which can be considered the main responsible for conferring the negative charge to the developed biomaterial systems. Moreover, collagen and chitosan will have a net charge dependent of the pH and, within the studied range, both would be positive to neutral. It is also important to refer that all systems showed similar surface zeta potential values, being the polymer concentration in each composition responsible for the small variations.

**Figure 8:**
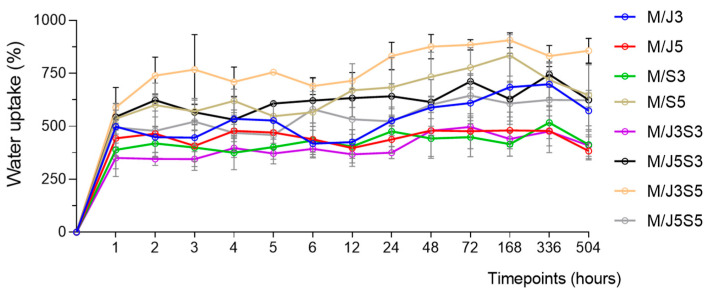
Assessment of the degree of swelling (measured as water uptake) in each membrane (M/J3 to M/J5S5) upon incubation in PBS solution for up to 21 days (504 h). Data are presented as mean ± standard error (n = 3).

The water uptake test was performed to appreciate the swelling ability (hydration property) of the developed membranes and evaluate the material structural stability during the experimental time (21 days). The collected data are shown in Figure 8.

All the developed membranes absorbed most of the water within the first hour (1 h), reaching a value that was not significantly altered for many of the membranes until the end of the experiment, which comprised 21 days. Despite the limitations of the study, namely the impact of the removal of water excess with filter paper or considering that only water uptake contributes to weight variation (thus neglecting eventual degradation or partial solubilization of material) [67,68], differences between membrane formulations could be observed. The ones that exhibited higher water uptake were M/J3S5, M/J5S3 and M/S5_,_ comprising values in the range of 600 to 850%, while on the opposite side were M/J5, M/S3 and M/J3S3 with values of about 400%. This can be related with the contents of shark collagen, with the membranes produced with higher amount of sCOL apparently showing higher capacity to absorb water, although other variables might be also playing a role. Moreover, these results are according to the obtained XPS analysis, which indicates the hydrophobic nature of the biomaterial surface by the presence of fewer carbon-bonded with functional groups at the surface. Indeed, this higher swelling capacity observed proves that all samples contain hydrophilic groups directed towards the interior of the structure. In fact, the presence of polar hydrophilic groups, such as -OH and/or -COOH, present in the biomaterial network, allow the bonding with the water molecules that increase their capacity to accumulate a higher percentage of water [69]. Additionally, the crosslinking density present on each polymeric structure (formed by chemical or physical agents) plays a significant role in providing an equilibrium state on the samples since the swelling capacity is contradicted by the elastic retraction force present in the polymeric network [70]. It is also important to note that the temperature of 37 °C during 21 days applied in this methodology do not compromise the structure of developed biomaterials, which are suitable to be used as an implantable material in the human body. Additionally, the proposed membrane scaffolds revealed no significant signs of degradation, indicating the desired stability even in long-term experiments, being possible after the test handling the membrane easily without the risk of breaking.

### 2.6. Thermal Characterization of Polymeric Membranes

**Figure 9:**
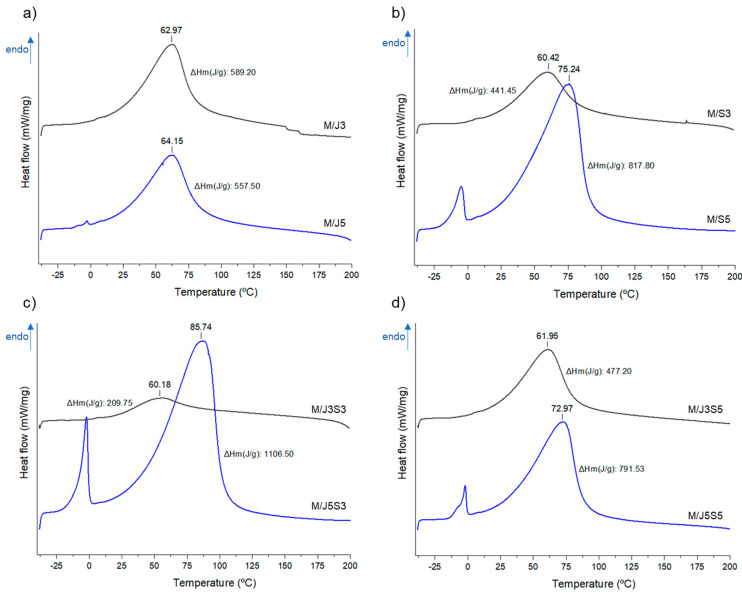
Differential scanning calorimetry (DSC) thermograms of the developed polymeric membranes M/J3 to M/J5S5 experiencing controlled heating from −40 to 200 °C: (**a**) M/J3 and M/J5; (**b**) M/S3 and M/S5; (**c**) M/J3S3 and M/J5S3 and (**d**) M/J3S5 and M/J5S5.

The differential scanning calorimetry (DSC) allows one to infer about the physico-chemical transformations induced by the controlled heating or cooling of the samples [71]. The developed membranes were submitted to a temperature range of −40 to 200 °C under an inert atmosphere, measuring the heat exchanges, with the resulting DSC thermograms being depicted in Figure 9.

In a first analysis, it is possible to visualize in some DSC thermograms (i.e., M/J5, M/S5, M/J5S3 and M/J5S5) the exhibition of a small endothermic peak that appears close to 0 °C. This phenomenon is associated with a melting phase transition (that occurs in temperatures below 0), which are promoted by a small fraction of free or absorbed water present in samples [72]. The second endothermic phenomenon observed in all biomaterial samples, registered within the range 60 to 85 °C, is related to the disruption of inter-/intra-chain hydrogen bonds that are present on the structural composition of each polymer and between them, essential to maintain the structural stability of the produced membranes [73]. Our results suggested that the membranes M/J5S3, M/S5 and M/J5S5 were the formulations with higher thermal stability when compared with the other samples, illustrated by the presence of the second endothermic peak at higher temperature and with higher associated enthalpy, i.e., as mentioned above, more energy is necessary to disrupt the hydrogen bonds. On the other hand, the biomaterials M/J3S3, M/S3 and M/J3S5 are those that present the lowest thermal stability. These differences can be associated with the type of collagen used and their concentration in each formulation: containing shark skin collagen at higher concentration provides higher structural matrix stability when compared with the formulations that included jellyfish collagen. Additionally, when analyzing the materials in the cooling step, no thermodynamic signals were observed, indicating the irreversibility of the thermal disruption of the polymeric matrices, at least within the timeframe herein studied.

**Figure 10:**
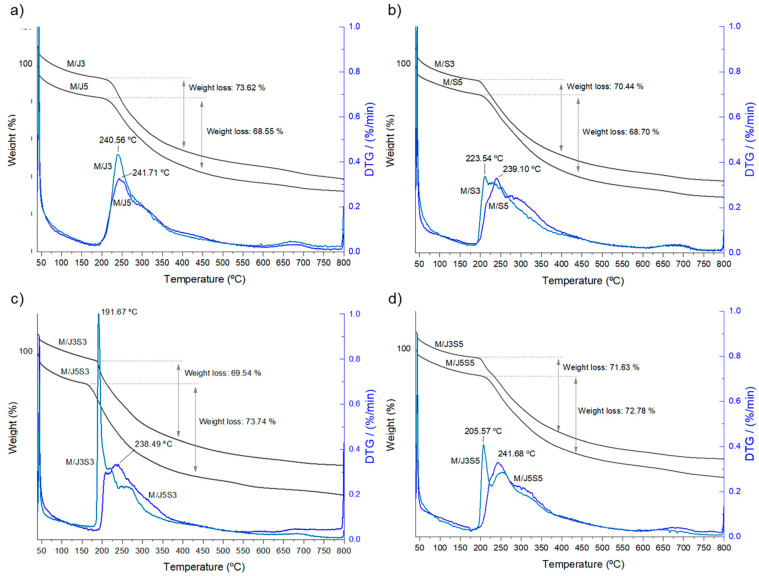
Thermogravimetric analysis (TGA) and derivative thermogravimetry (DTG—shown in blue) curves of the developed membranes in response to controlled heating from 40 to 800 °C: (**a**) M/J3 and M/J5; (**b**) M/S3 and M/S5; (**c**) M/J3S3 and M/J5S3; and (**d**) M/J3S5 and M/J5S5.

Further, a thermogravimetric analysis (TGA) was also performed to verify the weight loss experienced by the developed membranes upon heating (up to 800 °C, significantly above the range studied by DSC). The curves of weight loss and derivative thermogravimetry, or derivative weight loss (Δω/ΔT), are shown in Figure 10.

The TGA results can be divided into three principal temperatures ranges: zone A, which is between 50 to 200 °C, zone B, between 200 to 450 °C, and zone C, between 450 to 800 °C [71]. The curve in zone A corresponds to the evaporation of the residual physically absorbed water present in each sample, representing 11–15% of the total weight of the developed membranes and being observed only in some samples. The higher weight loss, corresponding to the thermal degradation of the organic compounds, was observed in zone B. In this stage, the samples have lost the internal structural network integrity (as observed in DSC analysis) and each component undergone combustion, resulting in a weight loss of approximately 70%. Lastly, the gradual low weight loss detected on zone C can be associated with some inorganic compounds that can be present on the samples experienced some chemical transformations, as well as some organic combustion still occurring. Additionally, the use of derivate of thermal analysis (DTG) allowed the determination of the temperature where the maximum rate of mass loss takes place, which in our samples was demonstrated to occur between 191 and 241 °C.

### 2.7. Mechanical Tests by Tensile Strength

**Figure 11:**
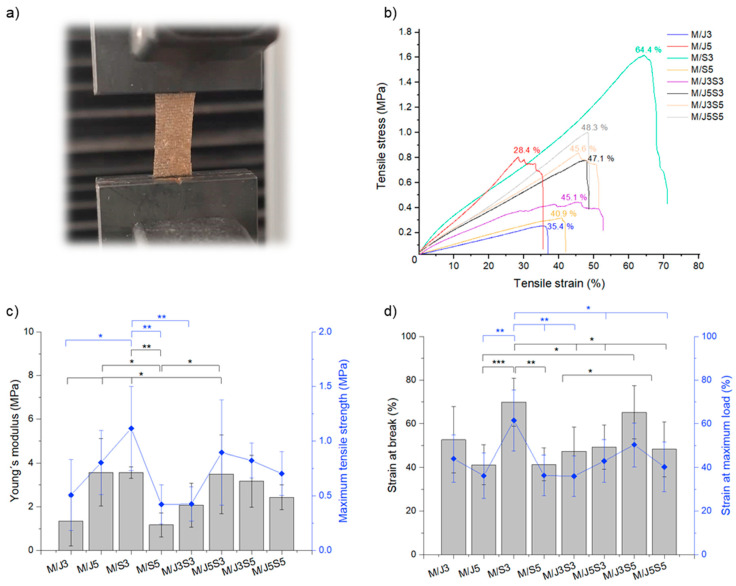
The mechanical properties of the marine biopolymers membranes (M/J3 to M/J5S5) addressed using uniaxial tensile testing. (**a**) Digital image of one condition (membrane M/J3) attached to Instron claws under load (representative of all samples); (**b**) Tensile stress–strain curves; (**c**) Young´s modulus and maximum tensile strength, and (**d**) strain at break and strain at maximum load. In graphics of (**c,d**), the error bars contain standard deviation (SD) from the mean values (not less than n = 5) and the symbols represent the statistical significance of * (*p* < 0.05), ** (*p* < 0.01), *** (*p* < 0.001) using one-way ANOVA with Tukey´s multiple comparisons test.

In order to evaluate the influence of polymeric concentration on the mechanical properties of the produced membranes, uniaxial tensile testing was carried out, as illustrated in Figure 11a, and the obtained results regarding stress–strain curves and determined parameters are collected in Figure 11b–d.

The mechanical properties are essential factors to consider in the manufacturing of scaffolds and membranes, especially if the target application is a tissue that is in constant excessive stress and strains, such as the hyaline cartilage tissue. Indeed, articular tissues are constantly subjected to stretching and contracting movements due to the biological mechanical forces exerted by the body locomotion [74]. To evaluate the mechanical properties of the developed scaffolds, the tensile and compressive tests are the most commonly used strategies. In the case of membrane samples, it is essential to measure the tensile that accesses the stiffness, tensile strength and maximum strain of the materials [75]. The stiffness is expressed as Young´s modulus, or modulus of elasticity, which defines the relationship between the tensile stress and the strain: the higher the value, the stiffer is the material. On the other hand, the tensile strength, also measured in MPa, indicates the maximum stress that the material to be tested can withstand before fracturing [76]. In general, the mechanical strength is identified by the resistance of the material in order to sustain its stable structural support and integrity during the implantation procedure and the therapeutic time, i.e., complete the full tissue regeneration [77]. In this order, it has been generally accepted that the intrinsic mechanical properties of the biomaterials should match with the native tissue, even because the known role of mechanotransduction of cellular fate and biomaterial effectiveness [78]. For example, in the case of native hyaline cartilage tissue, tensile Young´s modulus should be between 2 to 25 MPa, or possibly higher, and in normal loading conditions, the strains can reach up to 20–30% [79,80,81], while the native skin tissue has Young´s modulus values between 4 to 20 MPa with a strain at break around 35 to 115% [82]. Regarding the polymeric membranes herein studied, their Young´s moduli (Figure 11c) fall between 2 to 5 MPa and the strain at break (Figure 11d) was between 35 and 70%, being higher for samples M/S3, M/J5S3 and M/J3S5. Despite these values being in accordance with the requirements of both indicated tissues, they are closer to the lower limits, probably associated with the fact that the produced membranes were thin biomaterials relying on polymer entanglement supported by electrostatic interactions and hydrogen bonds (but not covalent crosslinking). In conclusion, comparing the results acquired with the modulus presented by both native tissues, it is possible to confirm that our biomaterials possess adequate mechanical properties to be used for the engineering of tissues as hard as articular cartilage since they can support the naturally mechanical stresses exercised in joints. Moreover, in future approaches, the developed membranes comprised interesting properties to be used, as well, in soft tissues such as skin; for example, by considering the exhibited elastic properties. The formulations that can be considered more adequate for this approach are M/S3 and M/S5, containing only shark collagen, understood as similar to type I collagen, which is the main constituent of the native skin tissues.

Additionally, in research previously published by our team [9,83,84] we performed biological in vitro analysis of biomaterials formulations comprising the marine polymers used herein, namely the evaluation of cell viability, cytotoxicity, DNA content, morphology and ATP activity, using chondrocyte (ATDC5) and fibroblast (L929) cell lines. Taking this into account, the present study aims to expand the scaffolding knowledge by exploring other processing methodologies of the same marine polymers for various purposes, in this case specifically to form membranes. In fact, studies with polymers for multiple purposes provides the advantage of investigating the best scaffold design for the final application and/or severity of the damage to the tissue. For instance, damaged cartilage tissue can be classified on a scale ranging from 1 (slight cartilage damage) to 4 (most severe cartilage damage) and the scaffolds can be designed according to this damage scale, providing a more adjusted therapy to each clinical case. Nevertheless, knowing that not only chemical composition, but also structural features can influence biological performance (regarding, for instance, the influence of morphological and mechanical properties), in vitro tests will be performed at later term to understand the potential of the herein proposed membranes to support viable chondrocytes and/or chondrogenic differentiation of stem cells.

## 3. Conclusions

In the present work, polymeric biomaterials were formulated based on natural/physical interactions between marine origin biopolymers, building membrane-like conformations through the use of a specifically designed mold comprising nylon mesh and 3D printed PLA. The combination of the polysaccharides squid chitosan and brown algae fucoidan with the proteins shark collagen and/or jellyfish collagen rendered polymeric matrices presenting good dimensional stability (and hand ability), flexibility, adequate swelling ability without compromising the original structure, uniform polymeric distribution, with slightly rough and hydrophobic surfaces and exhibiting mechanical properties similar to the ones observed in native cartilage tissue. The processing methodology was considered as eco-friendly according to the assessment of the *E*-factor, the effectiveness of which was dependent on the polymer concentration and the type of polymer. In particular, it was noticed that shark collagen contributed with more stability to the membranes than jellyfish collagen. Taking into account all the reported data, it is possible to point out the membranes M/S3 (produced by combination of equal volumes of 3% shark collagen solution, 3% squid chitosan solution and 10 % brown seaweed fucoidan solution) and M/J5S3 (5% jellyfish collagen, 3% shark collagen, 3% squid chitosan and 10% brown seaweed fucoidan) as the most attractive formulations, namely regarding their physical analysis, by exhibiting a significant water uptake needed to enable diffusion of nutrients and gases together with an ability to withstand higher mechanical forces, approximate to native tissue, being useful to cope with the constant forces exerted during the body locomotion.

Overall, this work shows that the developed marine polymeric membranes demonstrated promising performance for tissue engineering and biomedical fields, particularly as a thin biomaterial in the perspective of final application in human tissues, covering their damaged surface and promoting their regeneration. Therefore, all these membrane structures can be considered sustainable and could potentially be scaled up without a negative impact on the environment.

## 4. Materials and Methods

### 4.1. Materials

Collagen from jellyfish (*Rhizostoma pulmo*) (jCOL) was provided by Jellagen Pty Ltd. (UK). Collagen from blue shark (*Prionace glauca*) skin has been previously produced, as described by Diogo et al. [85]. Fucoidan from brown algae (*Fucus vesiculosus*) (aFUC) was acquired from Marinova (Cambridge, Australia, product: Maritech^®^ Fucoidan, FVF2011527), and used as received. Chitosan from squid pens (*Dosidicus gigas*) (sCHT) was produced and purified according to the patent number WO/2019/064231 [46]. In brief, the squid pens’ chitin was isolated and converted into chitosan using a single deproteinization and deacetylation step with an alkaline treatment, under a constant airflow of nitrogen (N_2_) at 75 °C for 2 h.

### 4.2. Solutions and Marine Membranes Preparation

**Figure 12:**
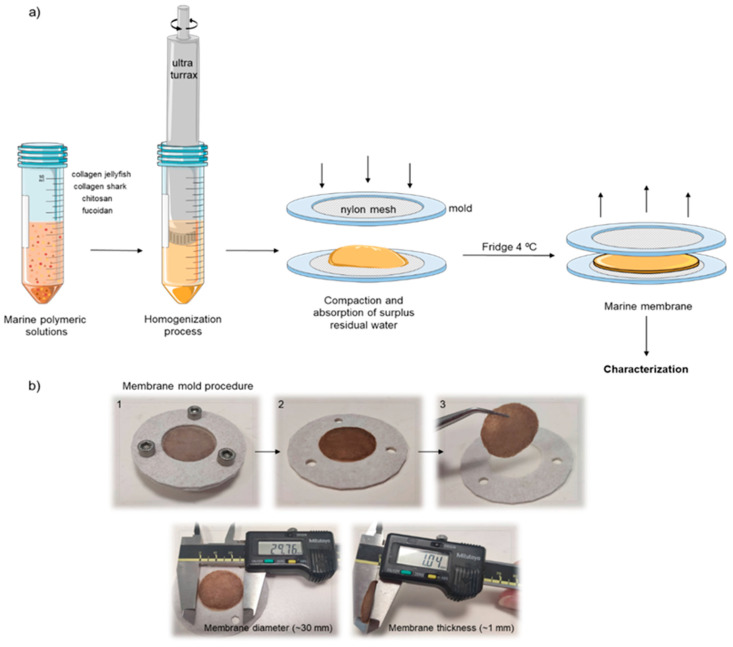
Preparation of marine biopolymers membrane. (**a**) Schematic representation of biomaterial-membrane formation process using a 3D printed PLA mold comprising a nylon mesh, where the biopolymers blend is placed and compacted while removing the excess of solvent; (**b**) Representative images of the molding procedure, the produced membranes and the respective dimensions.

Initially, both collagens and chitosan were separately solubilized in ammonium acetate buffer (0.15 M NH_4_OAc / 0.2 M AcOH) at pH 4.75 according to previously defined concentrations (30 and 50 mg/mL for collagens and 30 mg/mL for chitosan), while fucoidan was dissolved in ultra-pure water (100 mg/mL). Then, different marine polymeric solutions were mixed according to the formulations described in Table 2, and to guarantee the achievement of an appropriate homogenous mixture an overhead blender (Ultra-turrax T18, IKA, Staufen, Germany) was used, in low rotations to avoid bubbles, at 4 °C [86]. After that, each marine polymeric formulation was placed into a home-made cylindrical mold with a nylon mesh, previously produced by 3D printing with a polylactic acid (PLA) filament using Ender 3 Pro 3D printer (Creality, Shenzhen, China). Several filter paper strips were placed on top of each nylon mesh, to absorb the excess of solvents. Afterward, the molds were placed into the fridge at 4 °C for 3 days. During the molding time, the surplus solvent was removed, compacting the biopolymers and forming polyelectrolyte complexes by the action of natural cross-linking. A representative scheme of the procedure used to prepare the polymeric membranes is presented in Figure 12a.

To evaluate if our innovative process to manufacture the membranes is an environmental-friendly process, the green metrics environmental factor (*E*-factor) was used, being calculated according to the following Sheldon equation [29] (Equation (1)):

(1) $$E-\mathrm{factor}=\frac{\left(\Sigma m\;(\mathrm{raw}\;\mathrm{materials})+\Sigma m\;(\mathrm{reagents})+\Sigma m\;(\mathrm{solvents})\;-\;\Sigma m\;(\mathrm{products})\;\right)}{m\;(\mathrm{products})}$$

when is expressed the sum of raw materials, sum of mass of reactants, sum of mass of solvents and sum of mass of products, resulting in the ratio between the mass of total waste and the mass of products.

### 4.3. Marine Biopolymers and Membranes Characterization

To understand if the marine origin compounds chosen are adequate to be used in TERM and biomedical approaches, they were methodically previously characterized [8,83] to assess their natural properties in terms of physico-chemical properties, such as structure, stability, solubility, purity and being free of heavy metal elements, and were also evaluated to find if they have additional biological properties such as anti-oxidant activity. In fact, these preliminary characterizations are critical, allowing us to understand and predict their performance as a biomaterial, i.e., after polymeric reticulation, which is important for the present scaffolding study.

#### 4.3.1. Determination of Glycosylation by Glycoprotein/Carbohydrate Estimation in Collagen Samples

The glycosylation in collagen samples (from jellyfish and shark skin) was estimated using a glycoprotein carbohydrate estimation kit (Pierce™—Thermo Scientific, Waltham, MA, USA). For that purpose, 50 μL of each collagen sample (2.5 mg/mL) was placed in a 96 well-plate, and 25 μL of 10 mM sodium meta-periodate and 150 μL of 0.5% aldehyde detection reagent (Pierce™—Thermo Scientific, Waltham, MA, USA) were added and incubated for 1 h at room temperature. All samples and standards were tested in triplicate. Then, the optical absorbance was read at 550 nm in a microplate reader (Synergy HT, Bio-Tek Instruments, Winooski, VT, USA). Lysozyme and bovine serum albumin (BSA) were used as negative controls, while ovalbumin, human apotransferrin, fetuin and α1-acid glycoprotein were used as positive controls.

#### 4.3.2. Quantification of Total Collagen Concentration

The amounts of collagen present in marine collagen samples from jellyfish and shark, and in the developed biomaterial systems were determined by solubilization of 50 μg/mL of each sample in 1 M hydrochloric acid (HCl), and application of the enzymatic fluorimetric method using EnzyFluo™ collagen assay kit (ECOL-100) (Gentaur molecular products, Kampenhout, Belgium). The methodology was performed according to the manufacturer’s manual, and the fluorescence was read at λ_ex/em_ = 375/465 nm using a fluorescence spectrometer (JASCO, Tokyo, Japan) adapted with a microplate reader. Then, the concentration of collagen was calculated using the Equation (2):

(2) $$[\mathrm{collagen}]=\frac{F_{S\;}-F_{B\;}}{\mathrm{Slope}\;(\mu g/mL^{-1})}\times n$$

where F_s_ and F_B_ are fluorescence readings of the sample (F_s_) and blank (F_B_), and *n* is the sample dilution factor. The calibration curve slope was obtained after plotting the collagen concentration vs fluorescence of the standard.

#### 4.3.3. H Nuclear Magnetic Resonance (^1^H-NMR) Analysis

The atomic fingerprint of each of the marine biopolymers and subsequently the chitosan degree of deacetylation (DD) were determined by ^1^H Nuclear magnetic resonance (NMR) spectroscopy. The collagens and chitosan samples were solubilized (1%, *w*/*v*) in deuterium oxide (D_2_O) and Deuterium chloride (DCl) (Sigma-Aldrich, St. Louis, MO, USA), while the fucoidan samples were solubilized only in D_2_O, and then 1 mL of each solution was transferred to 5 mm NMR tubes. The ^1^H-NMR spectra (reported in ppm (δ)) were obtained by Bruker AVANCE 400 spectrometer, at 25, 45 and 60 °C using a resonance frequency of 400 MHz and a delay between pulses of 1 s. The data processing was determined using MestReNova Software 14.1 (Mestre-lab Research, Santiago de Compostela, Spain). The determination of chitosan DD (in%) was performed as described in the literature [87]. Briefly, using the data spectra of chitosan, the integrals of the CH_3_ of the N-acetyl group in GlcNAc (A_1_) and the remaining resonances from the ring positions were determined at the chemical shifts at ca. δ 2.9–4.0 ppm and ca. δ 2.0 ppm, A_1_ and A_2_, respectively. These values were then replaced as A_1_ and A_2_ in Equation (3).

(3) $$\mathrm{DD}\;(\%)=\left[1-\left(\frac{6\times A_{2}}{3\times A_{1}}\right)\right]\times \;100$$

#### 4.3.4. Ellman’s Test—Thiol Groups’ Quantification

The eventual presence of thiol groups (–SH) on the marine biopolymers and the developed biomaterial-membranes was quantified spectrophotometrically using Ellman’s method [57] that uses the reagent 5,5-dithio-bis (2-nitrobenzoic acid) (DTNB), also called Ellman’s reagent [88,89]. Briefly, all samples were dissolved in 100 mM DTNB with Dimethyl sulfoxide (DMSO) and left incubating protected from light, at room temperature, for 5 min. Then, the absorbance was read at 412 nm in a microplate reader (Synergy HT, Bio-Tek Instruments, Winooski, VT, USA). The quantity of thiol groups was estimated using a standard curve of L-cysteine (R_2_ = 0.98) and dH_2_O as a blank. The thiol concentration was calculated using the Equation (4):

(4) $$\mathrm{mM}\;\mathrm{of}\;\mathrm{Thiol}=\frac{\left(\Delta A_{412}\times \;1.1\;\mathrm{mL}\right)}{13.600\times \;0.4\;\mathrm{mL}}\times \;1000$$

where ∆A_412_ is the corrected absorbance value, and 13.600 is the molar extinction coefficient (cm^−1^ M) of the 5-thio-2-nitrobenzoate generated from Ellman’s reagent when reacting with the free thiol of the L-cysteine. The obtained results were expressed in nmol thiols/mg protein.

#### 4.3.5. Water Contact Angle Analysis

The water contact angle of the developed membranes was determined by the sessile drop method, using a contact angle meter (Goniometer OCA 15+, DataPhysics, Stuttgar, Germany) in association with an image processing system (SCA20 software, DataPhysics Instruments, Stuttgar, Germany). During every determination, a motor syringe was used to deposit a drop of water with 3 µL over the membrane surface. The images corresponding to these drops were recorded, and the contact angle was determined. Finally, the presented contact angles were calculated using measurements performed in the different membranes, in triplicate, at room temperature.

#### 4.3.6. Surface and Depth Profile Analysis by X-ray Photoelectron Spectroscopy (XPS)

For superficial sample composition, twelve (12) samples were fixed to the sample holder with double-sided carbon tape. The samples were analyzed using a Kratos Axis-Supra instrument equipped with aluminum K_α_ (Al-K_α_) monochromatized radiation at 1486.6 eV X-ray source, within ESCApe software. Photoelectron collection was performed from a take-off angle of 90º relative to the sample surface. The measurement in two distinct locations of each sample (n = 5) was performed in a Constant Analyser Energy mode (CAE) with a 160 eV pass energy for survey spectra and 20 eV pass energy for high-resolution spectra of C 1s, O 1s, Na 1s, N 1s, Si 2p Cl 2p, Ca 2p and S 2p. The binding energies (BEs) positions setting of the charge reference was equivalent to the C 1s hydrocarbon peak, the lower binding energy C 1s band at 285.0 eV [90]. The residual vacuum was maintained in the analysis chamber at around 7 × 10^−9^ torr.

Additionally, to analyze the compositive inside the samples, a relative depth profiling was accomplished using the same equipment. Blended membranes with distinct compositions were fixed to the sample holder with double-sided carbon tape and rastered over a 2 × 2 mm area at an angle of 90° to the surface. Sputtering occurred 35 times for 60 s intervals, using a PAH 16KeV Minibeam 5 ion gun. Regions’ spectra were acquired with a 20 eV pass energy for C 1s, O 1s, N 1s and S 2p, and the charge was corrected to the hydrocarbon bond that had binding energy of 285.0 eV. Atomic compositions were determined based on the region spectra peak areas provided in ESCAPE processing software.

Fucoidan sulfate group contents (such as -SO_3_) can be estimated using the basis of the sulfate (S) percentage that was determined by XPS analysis [91,92]. Moreover, these values can be used for the determination of sulfation degree in the fucoidan sample. For this purpose, two equations (Equations (5) and (6)) were employed [93].

(5) $$\mathrm{NSS}=\frac{C\;\%\;/\;12}{S\;\%\;/\;32}\;/\;6$$

(6) Degree of sulfation=1 / NSS

where NSS is the number of sulfate esters per monosaccharide, 12 and 32 are the atomic weight of carbon and sulfur, respectively, and the 6 corresponds to the number of carbon atoms in a sugar monomer (assuming that the monomers present in this polymer are hexoses).

#### 4.3.7. Scanning Electron Microscopy (SEM)

The surface morphology of each membrane was analyzed with a Nova NanoSEM 200 scanning electron microscope (SEM) (JSM-6010LV, JEOL, Tokyo, Japan). The samples were fixed on aluminum stubs using a mutual conductive adhesive tape and covered with gold using a Leica EM ACE600 (Leica microsystems, Austria) sputter coater.

#### 4.3.8. Surface Zeta (ζ) Potential Measurements

Zeta potential was measured using a SurPASS electrokinetic analyzer (Anton Parr) to assess the surface charge of each membrane during a pH range of 5.5 to 8. A fresh 0.1 M potassium chloride (KCl) (Sigma-Aldrich) solution was used as an electrolyte, and 0.05 M sodium hydroxide (NaOH) solution was used to increase the pH gradually on the KCl solution. For this analysis, each membrane was cut to obtain two identical circular sample pieces (*d* = 14 mm) and placed facing each other, with a gap between them of 100–110 μm submitted at a pressure of 400 mbar in bidirectional flow, according to the manufacturer’s recommendations. The measurements were performed in triplicate (n = 3) per condition and the results are expressed as mean ± standard deviation for the selected pH range.

#### 4.3.9. Swelling—Water Uptake Quantification

The water uptake abilities of developed membranes were studied by quantification of the respective weight variations. First, the dehydrated membrane weight (W_0_) was measured, and the material was then immersed in a pH = 7.4 solution of phosphate buffered saline (PBS) at 37 °C for 21 days. At different previously defined time points (1, 2, 3, 6, 12 h and 1, 3, 7, 14 and 21 days), the samples were withdrawn, soaked up with dried filter paper to remove the excess of solution, and weighed immediately (W_1_). All the assays were performed in triplicate (n = 3). Finally, the amount of solution absorbed by samples was calculated as percentage of the initial sample weight with the following Equation (7):

(7) $$\mathrm{Water}\;\mathrm{uptake}\;(\%)=(W_{1}-W_{0})\;/\;W_{0}\times \;100$$

#### 4.3.10. Differential Scanning Calorimetry (DSC)

Aliquots of the prepared membranes (~3 mg) were analyzed in a DSC Q100 equipment (TA Instruments, New Castle, DE, USA) using 40 μL aluminum pans covered with a suitable aluminum cover. DSC analysis was performed between −40 and 200 °C, at a heating rate of 10 °C/min, under nitrogen atmosphere using a flow rate of 50 mL/min. An empty aluminum pan was used as reference. All tests were performed twice.

#### 4.3.11. Thermogravimetric Analysis (TGA)

The weight variation of membranes aliquots (~10 mg) as a consequence of healting was determined using a TGA Q500 Thermogravimetric Analyzer (TA Instruments, New Castle, DE, USA). Experiments were performed at a heating rate of 10 °C/min, from 40 to 800 °C, under an air atmosphere. All tests were repeated once.

#### 4.3.12. Mechanical Tests by Tensile Strength

The mechanical properties of the developed membranes were addressed under uniaxial tensile tests, using an Instron 4505 universal mechanical testing equipment (Caerphilly, UK), equipped with a pneumatic BioPlus tensile grips system. All samples were cut, with rectangular shape and dimensions approximately of 30 mm (length) ✕ 5 mm (width) ✕ 1 mm (thickness), and a distance between grips of 25 mm. Furthermore, all samples were previously hydrated in PBS during 1 h (according to water uptake results). The tests were conducted at room temperature using a load cell of 50 N and a crosshead speed of 1 mm/min. The elastic modulus, maximum tensile strength, and maximum strain were calculated using the Bluehill Universal software. Six specimens per condition were tested, and the results are expressed in terms of mean ± standard deviation.

### 4.4. Statistical Analysis

Statistical analysis was performed by two-way ANOVA followed by Tukey’s post hoc test, using GraphPad Prism 8.0.1 (GraphPad Software, Inc., La Jolla, CA, USA). Differences between the groups were assessed considering a confidence level of 95%. In addition, the statistical analysis of surface zeta potential and mechanical tests results (no less than n = 5) were performed using the Kruskal–Wallis test, with Dunn’s comparison being used to determine statistical differences. The significance level between the groups were represented by symbols of * (*p* < 0.05), ** (*p* < 0.01), *** (*p* < 0.001), **** (*p* < 0.0001) and by *ns* (no significance). All data were presented as mean ± standard deviation (SD). As additional information, the equations present in this scientific paper were designed using the MathType 6.9 software (Design Science).

## Figures and Tables

**Table 1:** Average and standard deviation of the atomic concentration ratios between sulfur/carbon, sulfur/nitrogen and nitrogen/carbon on the surface of each biopolymers studied and on the developed biomaterials.

Samples	$\mathrm{Ratio}\frac{Sulfur(S)}{Carbon(C)}$	$\mathrm{Ratio}\frac{Sulfur(S)}{Nitrogen(N)}$	$\mathrm{Ratio}\frac{Nitrogen(N)}{Carbon(C)}$
jCOL	-	-	0.148 ± 0.030
sCOL	-	-	0.234 ± 0.010
sCHT	0.005 ± 0.000	0.050 ± 0.000	0.102 ± 0.014
aFUC	0.029 ± 0.000	2.101 ± 0.084	0.014 ± 0.000
M/J3	0.013 ± 0.001	0.325 ± 0.091	0.041 ± 0.006
M/J5	0.021 ± 0.007	0.341 ± 0.137	0.063 ± 0.003
M/S3	0.017 ± 0.005	0.339 ± 0.019	0.050 ± 0.020
M/S5	0.024 ± 0.001	0.585 ± 0.030	0.041 ± 0.004
M/J3S3	0.018 ± 0.001	0.356 ± 0.024	0.051 ± 0.003
M/J5S3	0.016 ± 0.000	0.302 ± 0.020	0.053 ± 0.001
M/J3S5	0.016 ± 0.000	0.220 ± 0.011	0.075 ± 0.004
M/J5S5	0.014 ± 0.001	0.265 ± 0.007	0.054 ± 0.006

**Table 2:** Membrane composition prepared by blending equal volumes of different marine origin biopolymer solutions (ratio of each biopolymer in the original solution and their percentage after biomaterial formation).

Polymeric Membrane Systems (100%)	Abbreviation	mg/mL of Polymer in the Original Solution			
		Collagen Jellyfish	Collagen Shark	Chitosan Squid Pens	Fucoidan Seaweed
jCOL_3_/sCHT/aFUC	M/J3	30; (18.8)	-; (0)	30; (18.8)	100; (62.4)
jCOL_5_/sCHT/aFUC	M/J5	50; (27.8)	-; (0)	30; (16.6)	100; (55.6)
sCOL_3_/sCHT/aFUC	M/S3	-; (0)	30; (18.8)	30; (18.8)	100; (62.4)
sCOL_5_/sCHT/aFUC	M/S5	-; (0)	50; (27.8)	30; (16.6)	100; (55.6)
jCOL_3_/sCOL_3_/sCHT/aFUC	M/J3S3	30; (15.8)	30; (15.8)	30; (15.8)	100; (52.6)
jCOL_5_/sCOL_3_/sCHT/aFUC	M/J5S3	50; (23.8)	30; (14.3)	30; (14.3)	100; (47.6)
jCOL_3_/sCOL_5_/sCHT/aFUC	M/J3S5	30; (14.3)	50; (23.8)	30; (14.3)	100; (47.6)
jCOL_5_/sCOL_5_/sCHT/aFUC	M/J5S5	50; (21.7)	50; (21.7)	30; (13.1)	100; (43.5)

mg/mL (*w*/*v*) of polymer concentration in the original solution; (% *w*/*w* of total polymer mass in the biomaterial-membranes).
